# Total lesion glycolysis in oral squamous cell carcinoma as a biomarker derived from pre-operative FDG PET/CT outperforms established prognostic factors in a newly developed multivariate prediction model

**DOI:** 10.18632/oncotarget.27857

**Published:** 2021-01-05

**Authors:** Gerrit Spanier, Daniela Weidt, Dirk Hellwig, Johannes K.H. Meier, Torsten E. Reichert, Jirka Grosse

**Affiliations:** ^1^Department of Cranio-Maxillofacial Surgery, University Hospital Regensburg, Regensburg, Germany; ^2^Department of Nuclear Medicine, University Hospital Regensburg, Regensburg, Germany

**Keywords:** FDG PET/CT, TLG, MTV, oral squamous cell carcinoma, prognostic value

## Abstract

Purpose: Retrospective study to investigate the impact of image derived biomarkers from [^18^F]FDG PET/CT prior to surgical resection in patients with initial diagnosis of oral squamous cell carcinoma (OSCC), namely SUV_max_, SUV_mean_, metabolic tumor volume (MTV) and total lesion glycolysis (TLG) of the primary tumor to predict overall survival (OS).

Materials and Methods: 127 subsequent patients with biopsy-proven OSCC were included who underwent [^18^F]FDG PET/CT before surgery. SUV_max_, SUV_mean_, MTV and TLG of the primary tumor were measured. OS was estimated according to Kaplan-Meier and compared between median-splitted groups by the log-rank test. Prognostic parameters were analyzed by uni-/multivariate Cox-regression.

Results: During follow-up 52 (41%) of the patients died. Median OS was longer for patients with lower MTV or lower TLG. SUV_max_ and SUV_mean_ failed to be significant predictors for OS. Univariate Cox-regression identified MTV, TLG, lymph node status and UICC stage as prognostic factors. By multivariate Cox-regression MTV and TLG turned out to be independent prognostic factors for OS.

Conclusions: The pre-therapeutic [^18^F]FDG PET/CT parameters MTV and TLG in the primary tumor are prognostic for OS of patients with an initial diagnosis of OSCC. TLG is the strongest independent prognostic factor for OS and outperforms established prognostic parameters in OSCC.

## INTRODUCTION

Oral squamous cell carcinoma (OSCC) is the sixth common malignancy in the world, with around 900,000 cases diagnosed per year. It accounts for 90% of all head and neck cancers. It is widely understood, that the evolution and progression of this cancer is a result of multiple stepwise alterations in cellular and molecular pathways within the squamous epithelium [1–5]. The main risk factors are tobacco and alcohol abuse and partly infection with human papilloma virus (HPV) [4, 6, 7]. Depending on patient and tumor factors the multidisciplinary therapeutic concept consists of surgery, radiation therapy, chemotherapy and targeted therapies. Despite these regimes and recent advancements in immune therapies the long-term prognosis is still poor, due to a high rate of locoregional recurrence and new malignant conversions [8–10]. Imaging enhances information beyond medical history and physical examination by assessing the tumor extent, possible bone infiltration and the presence of cervical nodal metastases in OSCC [11]. Regarding to this, computed tomography (CT) and magnetic resonance imaging (MRI) are the primary techniques for evaluation. However, there is evidence of a prognostic significance of metabolic biomarkers in positron emission tomography (PET) that cannot be visualized by CT and MRI [12].

The traditional and well established TNM staging system is based on the anatomical extent of tumor, metastases and certain histopathological features. Recently, in its eighth version, the American Joint Committee on Cancer (AJCC) introduced several new predictors like depth of tumor invasion and extranodal spread in cervical lymph node metastasis [13]. But it lacks specific biological and molecular properties of the tumor cells. An emerging hallmark of cancer cells amongst others is deregulated energy metabolism [14, 15].

Due to the high glucose utilization of many types of cancer PET with [^18^F]fluorodeoxyglucose (FDG) in combination with CT is well established in the diagnostic work-up of oncological patients. PET/CT in OSCC is commonly used at initial presentation to assess distant metastatic disease, to evaluate potential primary sites in the setting of an unknown primary cancer, to evaluate physiologic or pathologic activity within borderline cervical adenopathy and for post-treatment residual or recurrent disease [16–19]. FDG PET is superior to CT and MRI in the assessment of cervical, supraclavicular, and mediastinal lymph node involvement in patients with OSCC and in combination with CT or MRI it is supposed to be even more accurate. However, the improved accuracy of detection of distant metastasis and secondary tumors is the major advantage of FDG PET [20–22]. Moreover, FDG PET/CT provides accurate information on metabolic aspects especially in terms of tumor heterogeneity.

Despite its high sensitivity in the detection of cancer in the head and neck area, FDG PET/CT does not offer any advantage over contrast-enhanced CT or MRI in T-staging because the accurate assessment of tumor spread and the relationship between tumor and adjacent structures is difficult in unenhanced low-dose technique. FDG PET/MRI holds promise as an evolving modality in head and neck cancer [23]. On the other hand, PET/CT often enables a more precise contouring of solid tumors as part of therapy planning prior to radiation, which appears to result in better outcome of patients. This may be due to the fact that functional imaging is able to differentiate between malignancy and peritumoral edema. In some cases an early infiltration does not yet show a clear morphological correlate [24, 25].

Other applications in the post-treatment setting are the evaluation for local and distant tumor recurrence. Limiting factors are surgical and particularly radiation therapy-induced tissue alterations that cause increased uptake due to inflammation and can mimic tumor recurrence [19, 26–28]. In a prospective, randomized, controlled study, it could be shown that patients with locally advanced head and neck tumors can avoid surgery after chemoradiation without survival disadvantages if FDG PET/CT shows no pathologic glucose metabolism [19].

The standardized uptake value (SUV) is the typically used measure of glucose metabolism in clinical routine in FDG PET/CT. Reported as maximum (SUV_max_) or mean tumoral SUV (SUV_mean_), it does not fully reflect the metabolic properties of all the tumor cells. Lately this led to the introduction of new parameters for the analysis of FDG PET image data which quantify both anatomical and metabolic aspects of the entire tumor and/or metastases.

Metabolic tumor volume (MTV) is an index reflecting the size and extent of tissues with increased glucose metabolism, and total lesion glycolysis (TLG), the product of MTV and the SUV_mean_, comprehends both anatomical size and metabolic activity. Typically, there is a collinearity between the parameters pT-classification and MTV. Both measures turn out to be independent prognostic biomarkers in various solid malignancies [29–31]. In OSCC both can also be used to monitor therapeutic effects and to predict outcome [32, 33].

The purpose of this study was to investigate imaging biomarkers derived from pre-treatment FDG PET, including SUV_max_, SUV_mean_, MTV and TLG as potential predictors of OS in patients who underwent surgical resection of OSCC as primary treatment. Furthermore, to develop a multivariable prediction model for OS and to compare its prognostic value with established prognostic factors such as cervical lymph node status and UICC stage.

## RESULTS

### Patient characteristics

**Figure 1:**
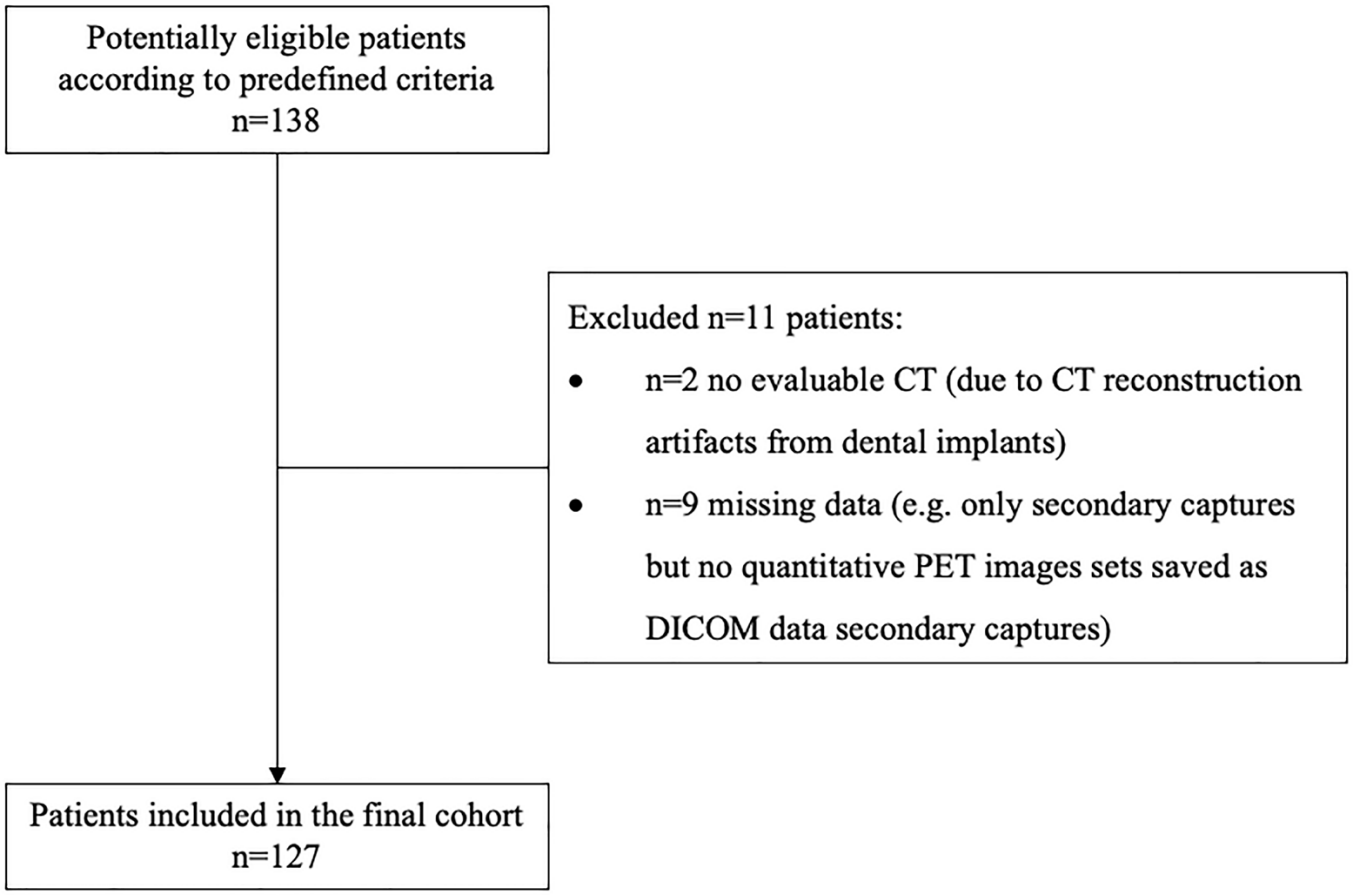
STARD (Standards for Reporting of Diagnostic Accuracy Studies) flow diagram.

Of the 138 consecutive patients, 11 patients were excluded according to the selection criteria as shown in Figure 1. Therefore, a total of 127 patients was included in this analysis: 93 men and 34 women with an age of 60 ± 10 years (range 35–83 years). The most frequent tumor site was the floor of the mouth. Smoking history was reported in 106 patients (83.5%), alcohol abuse in 93 patients (73.2%). Eighty patients (63%) had an advanced UICC stage III or IV, 60 patients had lymph node metastases (47.2%). Baseline clinical characteristics of enrolled patients are listed in Table 1.

**Table 1:** Clinical characteristics of the patients

Parameter	Patients
Sex	
Male	93 (73.2%)
Female	34 (26.8%)
Age (years)	
Range	35–83
Mean	60 ± 10
Smoking, *n* (%)	106 (83.5%)
Alcohol drinking, *n* (%)	93 (73.2%)
Smoking and alcohol drinking, *n* (%)	87 (68.5%)
Anatomical site, *n* (%)	
Buccal mucosa	10 (7.9%)
Upper alveolus and gingiva	7 (5.5%)
Lower alveolus and gingiva	29 (22.8%)
Hard palate	3 (2.4%)
Tongue	13 (10.2%)
Floor of mouth	65 (51.2%)
Cervical lymph node metastases	
Yes	60 (47.2%)
No	67 (52.8%)
UICC stage	
≤ II	47 (37%)
> II	80 (63%)
Adjuvant therapy	
None	58 (45.7%)
Radiotherapy	41 (32.3%)
Radio-chemotherapy	28 (22.0%)
Survival status	
Dead	52 (40.9%)
Alive	75 (59.1%)

### FDG PET derived parameters

**Figure 2:**
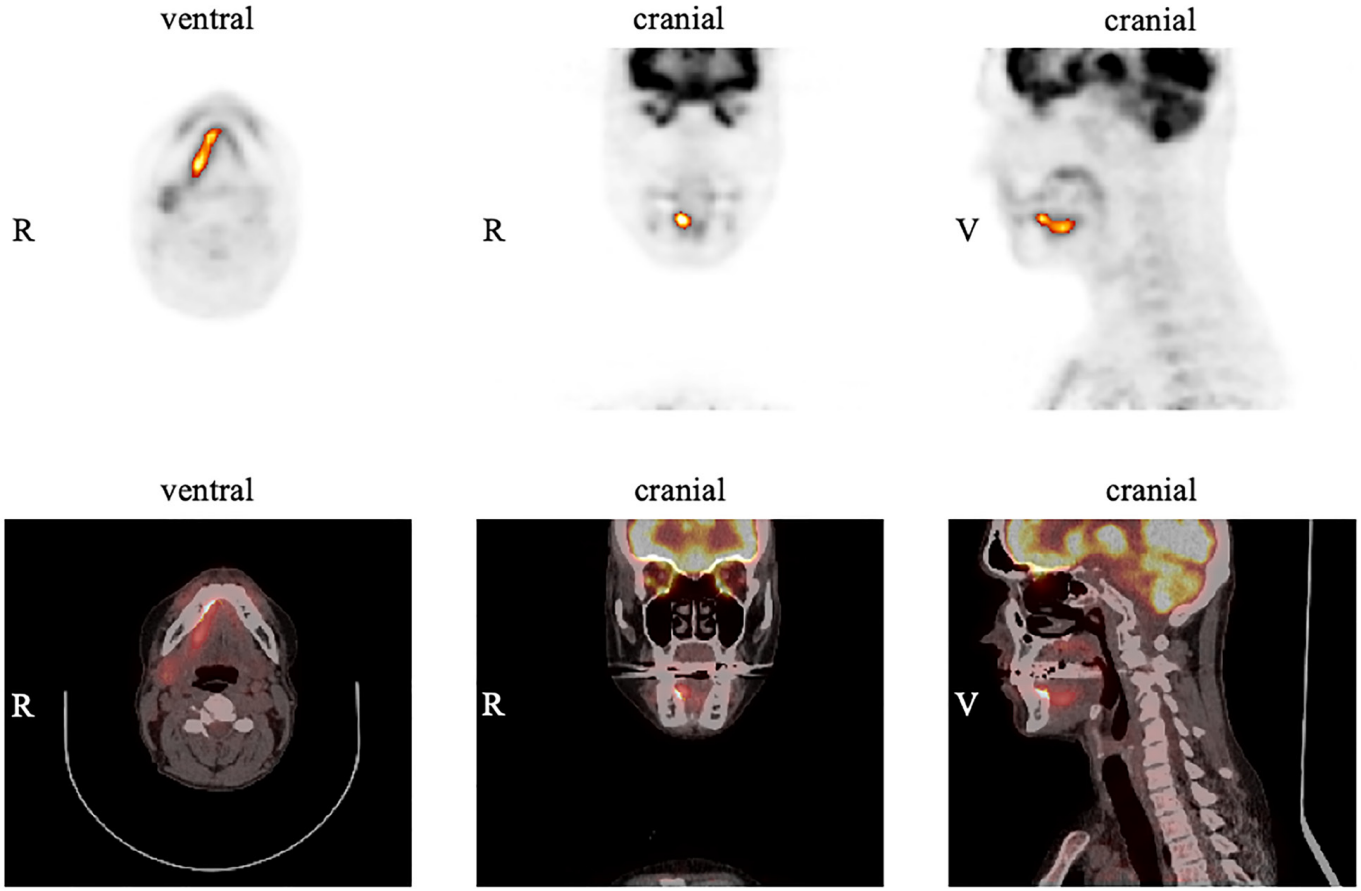
Example of measurements in FDG PET for the primary tumor using the software ROVER. The tumor is located in the right anterior floor of the mouth with an SUV_max_ of 12.8. Margins of the tumor were automatically delineated with a relative threshold of 41% SUV_max_ resulting in an MTV of 4.4 cm^3^ for the primary tumor.

A typical example of a patient with an OSCC in the floor of the mouth is demonstrated in Figure 2. Table 2 gives an overview for each of the parameters (SUV_max_, SUV_mean_, MTV, TLG) measured in the pre-operative FDG PET/CT of the primary tumor with the corresponding median, mean, standard deviation (SD), maximum, minimum, and interquartile ranges (IQR).

**Table 2:** Descriptive statistics of the image-derived biomarkers derived from FDG PET

Parameter	Mean ± SD	Median	Minimum	Maximum	IQR
**SUV_max_**	14.2 ± 7.1	12.8	3.9	38.2	8.8–17.8 = 9.0
**SUV_mean_**	8.5 ± 4.5	7.3	2.2	24.8	5.2–11.2 = 6.0
**MTV**	6.6 ± 5.5	5.3	1.4	43.6	3.1–8.0 = 4.9
**TLG**	60.2 ± 67.6	38.7	5.3	444.7	21.0–73.1 = 52.1

IQR: Inter-quartile range.

For the comparison between patients with and without histologically confirmed cervical lymph node metastases (N+), higher levels of SUV_max_ (16.8 ± 6.6 vs. 11.9 ± 6.7, *P* < 0.001), SUV_mean_ (10.1 ± 4.3 vs. 7.1 ± 4.1, *P* < 0.001) and TLG (75.8 ± 70.0 vs. 46.2 ± 62.3, *P* < 0.001) were found, whereas only a trend was observed to higher MTVs (7.1 ± 5.0 vs. 6.1 ± 5.9, *p* = 0.063). In the comparison between patients with UICC stage I+II and UICC stage III+IV, advanced stages exhibit higher SUV_max_ (16.7 ± 7.1 vs. 10.0 ± 4.6, *P* < 0.001), SUV_mean_ (10.1 ± 4.6 vs. 5.9 ± 2.7, *P* < 0.001), TLG (80.9 ± 77.4 vs. 25.1 ± 15.0, *P* < 0.001) and MTV (7.9 ± 6.4 vs. 4.4 ± 2.1, *P* < 0.001).

### Survival analysis

The median follow-up was 63 months with a known status for all patients at 36 months. During the follow-up period, 52 of 127 patients (40.9%) died. Median overall survival in the cohort was 83 months (CI: 60–106 months).

**Figure 3:**
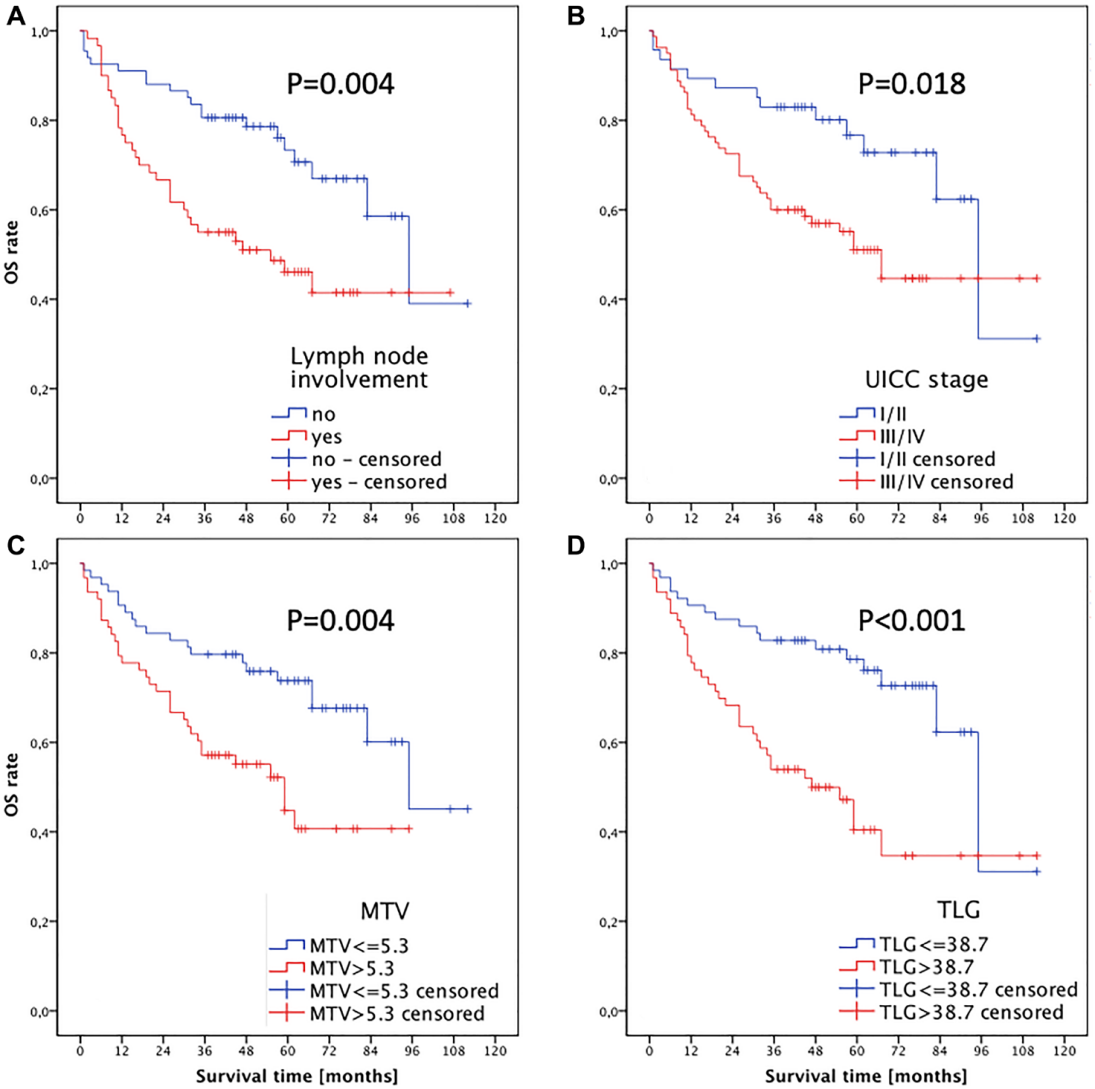
Kaplan-Meier analysis of overall survival for (**A**) cervical lymph node status, (**B**) UICC stage, (**C**) MTV and (**D**) TLG of the primary tumor.

Kaplan-Meier survival analysis showed a shorter OS of patients with lymph node metastasis (*P*(log-rank) = 0.004; as shown in Figure 3A) and with an UICC stage > II (*P*(log-rank) = 0.018; as shown in Figure 3B). In addition, a significant shorter median OS (59 months) could be observed in patients whose primary tumor had a MTV > 5.3 cm^3^ (*P*(log-rank) = 0.004), whereas a MTV of ≤ 5.3 cm^3^ was associated with an OS of 95 months (Figure 3C). Similarly, a higher TLG > 38.7 g was associated with shorter median OS (95 vs. 47 months, *P*(log-rank) < 0.001; as shown in Figure 3D). The PET parameters SUV_max_ and SUV_mean_ were not prognostic for OS.

In univariate analysis, lymph node status, UICC stage, MTV, and TLG were significant predictors for overall survival (Supplementary Table 1).

Due to the fact that TLG is the product of MTV times SUV_mean_ there is a strong correlation between MTV and TLG (*r* = 0.812, *P* < 0.001). Thus 2 separate models including either MTV (Model A, Supplementary Table 1) or TLG (Model B, Supplementary Table 1) were used for multivariate Cox regression analyses. Multivariate analyses showed that both MTV (HR 1.991 (CI 1.121–3.500), *P*(log-rank) = 0.019) and TLG (HR 2.808 (CI 1.563–5.047), *P*(log-rank) = 0.001) were prognostic factors for overall survival. According to its higher HR the TLG of the primary tumor results in the strongest independent prognostic parameter for OS.

## DISCUSSION

The aim of this retrospective study was to investigate the prognostic value of image derived biomarkers from [^18^F]FDG PET/CT performed prior to surgical resection in patients with initial diagnosis of OSCC. We focused on [^18^F]FDG metabolic parameters SUV_max_, SUV_mean_, MTV and TLG of the primary tumor to evaluate the impact on OS. In addition, the prognostic value of these parameters was compared with established clinical prognostic factors such as UICC stage and lymph node status.

Our study shows that MTV and TLG of the primary tumor are prognostic indicators of OS in patients at initial diagnosis of OSCC. Moreover, TLG is the strongest independent prognostic factor for OS and outperforms established prognostic parameters in OSCC like UICC stage and lymph node metastases. In contrast, PET parameters SUV_max_ and SUV_mean_ failed to be significant predictors of OS.

Different [^18^F]FDG PET scan derived parameters have been investigated in several tumor entities like lung cancer [34], head and neck cancers [32, 35, 36], solid tumors and in hemoblastoses/lymphomas [37–40].

In a retrospective study published by Higgins *et al.* in 2012, 88 patients with head and neck cancer were examined with [^18^F]FDG PET prior to definitive treatment by radiotherapy [41]. SUV_mean_ was determined in the primary tumor and lymph nodes. Patients with pretreatment tumor SUV_mean_ that exceeded the median value demonstrated inferior 2-year disease-free survival with 58% whereas a SUV_mean_ ≤ the median value of 15.4 was associated with a longer disease-free survival (82%, *p* = 0.03). Our results in OSCC before surgery cannot confirm the observations of the retrospective study of Higgins *et al.*. We found no association of SUV_mean_ and OS. In the univariate Cox regression, there was no difference in OS between patients with SUV_mean_ below or above the median of 7.3 (HR = 1.451, CI 0.835–2.521, *p* = 0.187). This discrepancy of the results may be related to several factors like different tumor entities (head and neck cancer located in oropharynx 66% of the patients versus OSCC) in different populations (radiotherapy candidates versus resected patients) investigated with different PET/CT scanners (GE Healthcare versus Siemens Healthineers). Whether SUV_mean_ is prognostic is best clarified in a prospective trial with well-defined populations.

The prognostic impact of SUV_max_ in head and neck cancer has been a subject of research for a number of years, with conflicting results [42–48]. Many studies have shown that SUV_max_ of the primary tumor is a significant prognostic factor for survival and an elevated SUV_max_ is associated with a poor clinical course [43, 45, 47, 48], whereas others did not support this association [49–51]. Alluri *et al.* conclude that these inconsistencies might be the result of the heterogeneity regarding tumor stage, tumor site, treatment modalities and the use of different outcome endpoints [52]. Moreover, technical differences between different PET scanners as well as imaging protocols may affect the comparability of quantitative PET measures between centers [53].

In their study Dibble *et al.* discussed the limited significance of the SUV_max_ [49], a parameter that represents the highest relative [^18^F]FDG accumulation, but is not representative for volume and biological activity of the entire tumor.

In our study, SUV_max_ of the primary tumor was not associated with OS in patients with OSCC. The different results of various studies in head and neck cancer suggest that the prognostic significance of the [^18^F]FDG-PET parameter SUV_max_ is questionable.

MTV, which indicates the volume of viable tumor defined by [^18^F]FDG uptake, is thought to be a more valuable predictor than SUV_max_ [54]. Several previous studies showed that MTV is a predictor for survival in patients with head and neck cancer. However, many of these studies included patients with tumors at different sites of the head and neck region which led to heterogeneity in the investigated cohort [49, 51, 54, 55]. Head and neck squamous cell carcinoma (HNSCC) are clinically heterogeneous entities that show variations in clinical behavior depending on the primary site. Studies that enroll patients with HNSCC of the entire head and neck region may be biased.

In the present study, we included only patients with OSCC prior to surgical resection to minimize the effect of heterogeneity. Ruy *et al.* also focused on patients with OSCC and found that pretreatment MTV is an independent prognostic factor for overall survival (*n* = 105, HR = 3.07; *p* = 0.001). They concluded that an MTV threshold of 3.0 mL may be useful to stratify the likelihood of survival and to predict occult metastases [50]. In contrast to our study, MTV was determined not only from the primary tumor but also included locoregional lymph node metastases.

Zhang *et al.* demonstrated the prognostic value of MTV (as defined by the primary tumor and local lymph node metastases) first in a study with a relatively small number of 80 patients with OSCC compared to the present study [56] and later validated their findings in a smaller cohort in 42 patients with OSCC [44]. An increase in MTV of 17.5 mL between the lower and upper tertials of the cohorts defined by MTV was associated with a 12.4 fold increase in risk of disease recurrence (*P* < 0.001) and a 11.2 fold increase in the risk of death (*P* < 0.05) [56], as they found during their comparably short follow-up with a mean duration of 1.9 years. Unfortunately, the exact thresholds for MTV derived from solely the primary tumor were not disclosed in the publications, so that a validation of their results in our patient cohort is not possible.

A higher MTV of on one hand tumor and involved lymph nodes (HR = 9.2, *P* < 0.05) as well as on the other hand the primary tumor alone (HR = 7.0, *p* = 0.0001) was associated with statistically significant increase in risk of death [44]. In their validation study MTV was the strongest prognostic parameter in multivariate Cox regression. Although less patients could be enrolled these results support our findings.

We did not include lymph nodes with increased FDG uptake into the MTV. Only tumoral MTV but not nodal MTV was identified as prognostic for survival as other studies found in similar patient populations [44, 51, 57].

Previous studies demonstrated that TLG is also a possible prognostic predictor for OS of patients with head and neck cancer. TLG, the product of MTV and SUV_mean_, combines the metabolic and volumetric information of FDG PET [58].

Dibble *et al.* showed that pretreatment TLG was an independent prognostic factor for OS in both univariate Cox regression (HR = 1.00, *p* = 0.006) and multivariate Cox regression (HR = 1.00, *p* = 0.02) in patients with oral and oropharyngeal SCC (*n* = 45) and may provide prognostic information in addition to AJCC stage [49]. However, the number of enrolled patients was much smaller compared to our study and the localization of the primary tumor was more heterogeneous.

In the aforementioned study of Ruy *et al.* in 105 patients with the initial diagnosis of OSCC, TLG could be established as a significant independent prognostic factor for OS (HR = 3.50, *p* = 0.002) [50]. In contrast to our study, TLG was measured in the primary tumor as well as in lymph node metastases.

These published studies demonstrate the prognostic value of MTV and TLG in patients with OSCC and confirm the results of our study, where TLG is not only an independent prognostic parameter, but the strongest predictive parameter for OS with respect to all investigated potential prognostic parameters (HR = 2.808, *p* = 0.001).

However, the differences between our study and the other studies must be taken into account. Results have to be interpreted carefully in the context of the retrospective study design. The number of patients enrolled, and the period of follow-up were limited, albeit higher than in many previously published studies. Due to poor tumor delineation in native low-dose technique volumetric data of CT were not included.

HPV infection is no relevant prognostic parameter in current TNM staging and was not assessed in this study. In addition, HPV status has no effect on the prognosis of OSCC patients compared to other HNSCC locations. Several authors rate the influence of HPV-triggered carcinogenesis in OSCC as minor [59, 60].

Transparent reporting of prediction models including thresholds used for the definition of subgroups is required for reproducibility results and validation of previous observation. Unfortunately, several publications cited here fail to meet these criteria which impedes verification. We have oriented to the TRIPOD standard for our report [61].

In conclusion, TLG and MTV reflect the metabolic burden of the primary tumor more precise than SUV_max_ and SUV_mean_ and may have superior prognostic value in patients with OSCC compared to other FDG PET parameters und established prognostic clinical parameters. Because OSCC patients with a higher MTV and TLG of the primary tumor prior to treatment have poor OS, FDG PET/CT may be useful for risk stratification. Thus, the measurement of these metabolic parameters could be helpful to select treatment and follow-up strategies such as more extensive surgery and aggressive adjuvant chemoradiation.

A recent meta-analysis by Creff *et al.* evaluated 36 studies about the prognostic significance of preoperative FDG-PET/CT parameters in HNSCC and suggested them as valuable biomarkers. With SUV_max_ as the most commonly measured factor, they confirmed that the volumetric parameters (MTV, TLG) presented a higher prognostic value for several primary endpoints. Six studies focused on the oral cavity with a range of 28-148 participants (median 75.5) and the results were in line with our data [62].

These results need to be confirmed in a retrospective study using an independent data set or even better validated in a prospective study. Radiomics is an emerging and promising approach to research in medical imaging. It is contemporary in transition to clinical practice and needs to overcome several obstacles in terms of standardization, validation and software integration for a feasible clinical workflow.

In the future, image-derived biomarkers from FDG PET/CT may be implemented in risk stratification to manage therapy strategies in patients with OSCC.

## MATERIALS AND METHODS

### Patients and clinical data

In this retrospective study, all subsequent patients with newly diagnosed OSCC between 2006 and 2013 were included who underwent an [18F]FDG PET/CT scan in the Department of Nuclear Medicine (University Hospital Regensburg, Regensburg, Germany) for initial staging prior to surgery. Only patients without neoadjuvant treatment were included. Patients with proven distant metastasis at the time of staging mostly received systemic therapy (depending on the decision of the tumor conference combined with local radiotherapy) and were not enrolled in the study. All included patients underwent surgical resection of the primary intraoral lesion to negative histopathologically proven margins and neck dissection based on the clinical and imaging findings in the Department of Cranio-Maxillofacial Surgery (University Hospital Regensburg, Regensburg, Germany). A total of 127 patients met the criteria and were enrolled in the study.

All patients were staged accordingly to the Union for International Cancer Control (UICC) guidelines in its seventh edition. Patient data were obtained by review of the medical records containing documentations of structured patient interviews prior to surgery. The data included age, weight and height at diagnosis, sex, history of smoking and drinking habits, tumor site, tumor-node-metastasis (TNM) stage, tumor grade and resection status.

Adjuvant treatment was based on the recommendation of the multidisciplinary tumor board, and radiotherapy and systemic therapy was performed accordingly. Disease progression was defined as local disease recurrence or distant metastasis by radiologic evidence with clinical correlation or histologic confirmation with biopsy. Data concerning OS were obtained from medical records and the clinical cancer register Regensburg (Germany).

Approval from the local ethics committee of the University Hospital Regensburg was obtained (reference number 16-104-0191), and this retrospective study was performed in accordance with all relevant guidelines and regulations.

### Imaging

[^18^F]FDG PET/CT imaging was performed using a Biograph 16 PET/CT scanner (CTI-Siemens, Erlangen, Germany) that consists of a PET detector with an axial and transaxial field-of-view of 162 mm and 585 mm and a 16-slice multidetector CT (0.5 s per revolution).

After a fasting period of at least 6 h, 3 MBq [^18^F]FDG per kilogram body weight were injected intravenously (338 ± 32 MBq). The patients’ blood glucose level was below 150 mg/dL (8.32 mmol/L). Patients were advised to stay in a quiet lying position to minimize muscular [^18^F]FDG uptake. In order and to keep potential tracer accumulation in brown fat tissue to a minimum, warming blankets were used to avoid freezing of the patients. Prior to scanning patients were instructed to void the bladder and to remove all metal parts.

After a waiting period of about 60 min post-injection (74 ± 19 min), the PET/CT acquisition was performed with elevated arms to acquire images of the trunk (six to eight overlapping bed positions with 3 min of PET acquisition time each depending on the patient size) followed by dedicated images of the head and neck with the arms down (two overlapping bed positions with 5 min per bed position). The same area was covered by a low-dose CT scan (tube current 50 mAs, tube voltage 120 kVp), respectively. No oral or intravenous contrast agents were used.

After correction for attenuation, decay, scatter, and random coincidences, and iterative reconstruction using the ordered subsets expectation maximization algorithm (OSEM) with 4 iterations and 8 subsets PET images (slice thickness 5 mm) were scaled to allow SUV measurements. PET and CT images were checked for breathing/motion artifacts.

### Image analysis

Two experienced observers (JG, DW) reviewed the reconstructed and attenuation-corrected [^18^F]FDG PET/CT images visually on the workstation, with reference to maximum intensity projection, PET/CT fusion and CT images, until consensus was reached. The observers were blinded to clinical parameters and patient outcome.

Spherical or ellipsoidal region of interest (ROI) was placed over the hypermetabolic primary lesions visible on PET images to obtain a three-dimensional coverage of the PET positive tumor on axial, sagittal and coronal projections. If necessary, the tumor was manually delineated using the corresponding CT images.

SUV_max_, SUV_mean_, MTV and TLG of the primary tumor were measured and automatically calculated using ROVER (ABX, Radebeul, Germany). For calculation of the MTV the margins of the tumor were defined using a relative threshold of 41% of the SUV_max_. TLG was defined as MTV × SUV_mean_ [58].

### Statistical analysis

All statistical analyses were performed using IBM SPSS Statistics Version 24.0 (IBM Corp., Armonk, N.Y., USA). Continuous variables are expressed as mean with standard deviation (SD) or median with ranges, and categorical variables as counted number with portions in percentages. Deviations from normal distribution were visually inspected by graphic analyses, box plot and histograms as well as analyzed by Kolmogorov-Smirnov and Shapiro-Wilk test. Nonparametric Mann-Whitney-*U* test was used for comparisons of continuous measures between groups.

OS was defined as time from FDG PET/CT until death of any cause or censored at last patient contact. The period for the survival analysis was planned for a minimum follow-up of 36 months for all patients. OS curves were calculated using the Kaplan-Meier method. The log-rank test was used to compare survival between subgroups defined by potentially prognostic parameters at a significance level of *P* < 0.05. Subgroups of patients with lower and higher SUV_max_, SUV_mean_, MTV and TLG were defined by dichotomization using the respective median.

The Cox proportional-hazards model was used to evaluate prognostic variables for univariate and multivariate prediction of OS. Multivariate analysis was carried out by Cox regression analysis with backward stepwise exclusion. Hereby we started with the full set of variables with stepwise exclusion of the variable with the largest *P*-value keeping only variables with a significance level of *P* < 0.10. We repeated this process until no variable in the model had a *P*-value greater than or equal to the significance level in order to identify prognostic factors in respect of the primary endpoint OS. The estimated hazard ratio (HR) and 95% confidence intervals (CI) were calculated.

Correlation coefficients were calculated and tested for significance according to Spearman. All tests were two-sided, and *P* < 0.05 was considered statistically significant.

### Ethical approval

This research study was conducted retrospectively from data obtained for clinical purposes. Approval from the local ethics committee of the University Hospital Regensburg was obtained (reference number 16-104-0191), and this retrospective study was performed in accordance with all relevant guidelines and regulations.

## SUPPLEMENTARY MATERIALS





## Abbreviations

AJCC: American Joint Committee on Cancer
CI: Confidence interval
CT: Computed tomography
FDG: [^18^F]fluorodeoxyglucose
HPV: Human papilloma virus
HR: Hazard ratio
IQR: Interquartile ranges
MBq: Megabecquerel
MRI: Magnetic resonance imaging
MTV: Metabolic tumor volume
OS: Overall survival
OSCC: Oral squamous cell carcinoma
PET: Positron emission tomography
SCC: Squamous cell carcinoma
SD: Standard deviation
SUV: Standardized uptake value
SUV_max_: Maximum tumoral standardized uptake value
SUV_mean_: Mean tumoral standardized uptake value
TLG: Total lesion glycolysis
TNM: Tumor-node-metastasis
UICC: Union for International Cancer Control
