# Supplementary material for: Total lesion glycolysis in oral squamous cell carcinoma as a biomarker derived from pre-operative FDG PET/CT outperforms established prognostic factors in a newly developed multivariate prediction model
Source: Oncotarget. 2021 Jan 5;12(1):37–48. doi: 10.18632/oncotarget.27857 (PMC7800778; doi:10.18632/oncotarget.27857)
Supplement: Supplementary file 1 [file oncotarget-12-37-s001.pdf]

# Total lesion glycolysis in oral squamous cell carcinoma as a biomarker derived from pre-operative FDG PET/CT outperforms established prognostic factors in a newly developed multivariate prediction model

## SUPPLEMENTARY MATERIALS

**Supplementary Table 1: Correlation of potential prognostic parameters and overall survival (OS).**
See Supplementary Table 1
