# Supplementary material for: Total lesion glycolysis in oral squamous cell carcinoma as a biomarker derived from pre-operative FDG PET/CT outperforms established prognostic factors in a newly developed multivariate prediction model
Source: Oncotarget. 2021 Jan 5;12(1):37–48. doi: 10.18632/oncotarget.27857 (PMC7800778; doi:10.18632/oncotarget.27857)
Supplement: Supplementary file 2 [file oncotarget-12-37-s002.docx]

**Supplementary Table 1: Correlation of potential prognostic parameters and overall survival (OS)**

| **Risk** | **Groups** | **Median survival [months]** | **P**  **Log-rank** | **Univariate Cox regression**  **B coefficient**  **(P value)** | **Univariate Cox regression**  **Hazard ratio (CI)** | **Multivariate Cox regression**  **Model A**  **B coefficient**  **(P value)** | **Multivariate Cox regression**  **Model A**  **Hazard ratio (CI)** | **Multivariate Cox regression**  **Model B**  **B coefficient**  **(P value)** | **Multivariate Cox regression**  **Model B**  **Hazard ratio (CI)** |
| --- | --- | --- | --- | --- | --- | --- | --- | --- | --- |
| Sex | Male (*n* = 93)  Female (*n* = 34) | 83  not reached | 0.641 | 0.149  (0.643) | 1.160  (0.619-2.175) | - | - | - | - |
| Age ≥ 60 years | Yes (*n* = 60)  No (*n* = 67) | 83  67 | 0.583 | 0.151  (0.586) | 1.163  (0.675-2.006) | - | - | - | - |
| Smoking history | Yes (*n* = 106)  No (*n* = 11) | 83  not reached | 0.312 | 0.411  (0.318) | 1.509  (0.673-3.379) | - | - | - | - |
| Drinking history | Yes (*n* = 93)  No (*n* = 34) | 95  67 | 0.645 | 0.147  (0.648) | 1.158  (0.617-2.172) | - | - | - | - |
| Smoking and Drinking | Yes (*n* = 87)  No (*n* = 40) | 83  67 | 0.442 | 0.235  (0.445) | 1.264  (0.692-2.309) | - | - | - | - |
| Cervical lymph node metastases | Yes (*n* = 60)  No (*n* = 67) | 55  95 | 0.004 | 0.804  (0.005) | 2.234  (1.274-3.915) | 0.683  (0.017) | 1.980  (1.121-3.500) | dropped  (0.165) | dropped |
| Initial UICC stage | I/II (*n* = 47)  III/IV (*n* = 80) | 95  67 | 0.018 | 0.739  (0.021) | 2.095  (1.116-3.933) | dropped  (0.942) | dropped | dropped  (0.756) | dropped |
| Adjuvant Treatment | Yes (*n* = 69)  No (*n* = 58) | 67  95 | 0.068 | 0.522  (0.073) | 1.685  (0.952-2.980) | - | - | - | - |
| SUV_max_ > 12.8 g/mL | Yes (*n* = 65)  No (*n* = 62) | 67  83 | 0.226 | 0.336  (0.231) | 1.400  (0.808-2.425) | - | - | - | - |
| SUV_mean_ > 7.3 g/mL | Yes (*n* = 63)  No (*n* = 64) | 67  83 | 0.182 | 0.372  (0.187) | 1.451  (0.835-2.521) | - | - | - | - |
| MTV > 5.3 mL | Yes (*n* = 63)  No (*n* = 64) | 59  95 | 0.004 | 0.815  (0.005) | 2.260  (1.277-3.999) | 0.689  (0.017) | 1.991  (1.120-3.541) | excluded | excluded |
| TLG > 38.7 g | Yes (*n* = 63)  No (*n* = 64) | 47  95 | < 0.001 | 1.033  (0.001) | 2.808  (1.563-5.047) | excluded | excluded | 1.033  (0.001) | 2.808  (1.563-5.047) |

SUV: standardized uptake value. MTV: Metabolic tumor volume. TLG: Total lesion glycolysis. X: excluded by stepwise Cox regression. As MTV and TLG are strongly correlated (r=0.812, *P* < 0.001) 2 models (A including MTV, B including TLG) were analyzed by multivariate Cox regression analysis. Model B showed that TLG is the only independent prognostic parameter with significant OS difference for patients with TLG>38.7 g in the primary tumor
